# The prognostic significance of CD117-positive mast cells and microvessel density in colorectal cancer

**DOI:** 10.1097/MD.0000000000038997

**Published:** 2024-07-19

**Authors:** Betul Bolat Kucukzeybek, Yelda Dere, Aysegul Akder Sari, Irfan Ocal, Emel Avcu, Ozcan Dere, Aylin Orgen Calli, Cigdem Dinckal, Mine Tunakan, Yuksel Kucukzeybek

**Affiliations:** a Department of Pathology, Izmir Katip Celebi University Ataturk Training and Research Hospital, Izmir, Turkey; b Department of Pathology, Mugla Sitki Kocman University, Faculty of Medicine, Mugla, Turkey; c Department of Surgery, Mugla Sitki Kocman University, Faculty of Medicine, Mugla, Turkey; d Department of Medical Oncology, Izmir Katip Celebi University Ataturk Training and Research Hospital, Izmir, Turkey.

**Keywords:** colorectal neoplasm, mast cells, microvascular density, prognosis, survival

## Abstract

The prognostic significance of angiogenesis has been demonstrated in various types of cancer. However, in colorectal cancer (CRC), there are conflicting results regarding the relationship between angiogenesis and clinical-histopathological prognostic factors. Mast cells are immune system cells found in the inflammatory microenvironment; their role in carcinogenesis and prognosis remains unclear although they are considered to cause cancer development and progression. The present study aims to evaluate the prognostic significance of mast cell accumulation and angiogenesis assessed by microvessel density (MVD) in patients with CRC. Patients who underwent curative resection and who were not treated with neoadjuvant chemotherapy were included. The anti-CD34 antibody and anti-CD117 antibody were utilized for the immunohistochemical assessment of MVD and the mast cell count (MCC) in the tissue samples, respectively. The relationship between MCC, MVD, survival and clinical-histopathological prognostic factors were evaluated. A total of 94 patients were enrolled to the study. In a median 49-month follow-up, 65 patients (69.1%) died. The 5-year disease-free survival was 61.1% and 31.3% for the group with CD34 < 18.3% and CD34 > 18.3%, respectively (*P* = .001). The same groups presented 5-year overall survival rates of 77, 1% and 51, 4%, respectively (*P*, .012). The MVD was found to be associated with the pathological T stage, lymph node metastasis and distant metastasis (*P* < .05). Although the MCC was positively correlated with MVD, there was no association between the MCC and clinical-histopathological prognostic factors. MVD-assessed angiogenesis was significantly related to survival and the clinical-histopathological prognostic factors in patients diagnosed with CRC.

## 1. Introduction

Colorectal cancer (CRC) is the third most frequent cause of cancer-related death in both women and men.^[1]^ The most crucial prognostic factor for CRC is its pathological stage. In addition to the pathological stage, histological grade, vascular invasion, and perforation-obstruction are other prognostic factors.^[2]^ In operated CRC, adjuvant chemotherapy is administered depending on the stage of the disease. However, recurrence or metastasis is observed despite the treatments applied.^[3]^ In a surgical approach to CRC, adjuvant chemotherapy is administered in accordance with the stage of the disease. Nevertheless, despite the administration of various treatments, recurrence or metastasis is observed.

Tumor angiogenesis refers to the formation of new blood vessels out of those present in or around the tumor and it is necessary for tumor growth, invasion, and metastasis.^[4]^ Folkman^[5]^ reported that mast cells are attracted through chemotactic factors synthesized by tumor cells, and mast cells may release proangiogenic factors. The effect of angiogenesis on tumor progression has been proven in many studies. Microvessel density (MVD) is a popular histological measurement technique for angiogenesis. MVD has been demonstrated to be an independent prognostic factor for a multitude of tumors, including breast cancer, prostate cancer, and lung cancer.^[6–8]^ However, investigations examining the correlation between angiogenesis, as assessed by MVD, and prognostic factors in CRCs have yielded disparate outcomes. Some studies have indicated that increased angiogenesis is associated with aggressive behavior, while others have suggested that it is linked to prolonged survival.^[9–11]^ Additionally, there are studies that have not reported a correlation between angiogenesis and prognosis.^[12,13]^

Mast cells are components of the immune system and are located within the inflammatory microenvironment. Despite their potential to contribute to the development and progression of cancer, the precise role of mast cells remains uncertain.^[14]^ The existing literature contains conflicting evidence regarding the involvement of mast cells in tumorigenesis and tumor progression. Nevertheless, it has been demonstrated that mast cells may play a role in inflammation-associated colorectal carcinogenesis.^[15]^ Conversely, they have been demonstrated to play a protective role in the early stages of intestinal tumorigenesis.^[16]^ It has been demonstrated that the accumulation of mast cells is associated with the rapid growth and invasion of certain tumors, including breast, lung, and prostate carcinomas.^[17–19]^ Additionally, there is a lack of consensus regarding the relationship between mast cell density and prognosis in colorectal carcinomas.^[9,20–22]^

The objective of the present study was to retrospectively investigate the relationship between c-Kit-positive mast cell count (MCC) and MVD as well as the relationship between c-Kit-positive MCC and angiogenesis assessed by MVD in patients with CRC, with a focus on prognostic clinicopathological data.

## 2. Methods

### 2.1. Patients and tissue samples

This study was approved by the local ethics committee of Izmir Katip Celebi University Ataturk Training and Research Hospital. The study included patients diagnosed with CRC between January 2010 and December 2011 who underwent curative resection and did not receive neoadjuvant chemotherapy. Patient demographics and survival data were obtained from follow-up files at the medical oncology outpatient clinic. Staging was performed by the TNM classification. Clinicopathological data, including tumor stage, size, patient age, lymph node status, presence of lymphovascular invasion, recurrence or metastasis, and histological grade, were obtained from the patient files. Disease-free survival (DFS) was defined as the period from diagnosis until relapse, metastasis, death, or the last follow-up date. Overall survival (OS) was defined as the period from the date of diagnosis to the time of death due to any cause.

### 2.2. Immunohistochemical method

Immunohistochemical staining was conducted using the streptavidin-biotin-peroxidase method, with 4-micron sections extracted from tumor-containing blocks embedded in paraffin and fixed in formalin. The sections were stained with CD34 (monoclonal mouse antibody, clone QBEnd10, dilution 1:40; Dako, Denmark) and CD117 (clone polyclonal rabbit, dilution 1:400; Dako). Formalin-fixed, paraffin-embedded tissue sections of 4 μm from each representative block were placed onto polylysine-coated slides for immunohistochemical staining with CD34 and CD117. Immunohistochemistry was performed by using the standard streptavidin-biotin-peroxidase complex (Leica Microsystems, Buffalo Grove, IL) method using the Leica Bond-Max autoimmunostainer with Bond Polymer Refine Detection Kits and heat-induced epitope retrieval pH 8.0 (Bond max ER2 [EDTA] solution; Melbourne, Australia) for 15 minutes autoimmunostainer with Bond Polymer Refine Detection Kits and heat-induced epitope retrieval pH 8.0 (Bond max ER2 [EDTA] solution; Melbourne, Australia) for 15 minutes. Two pathologists counted CD117-positive mast cells in 3 separate areas of the tumor stroma, adjacent mucosa, and invasive side of the tumor at 40x magnification. MVD assessment was conducted using anti-CD34 antibody. For MVD assessment, the stained sections were initially scanned at low magnification (40× and 100×) in order to identify the areas with the highest number of microvessels within the tumor (hot spots). The 3 most vascularized areas were counted within a 40× microscopic field, and the mean microvessel count was noted as the MVD. Any cell exhibiting brown cytoplasmic staining was considered a single countable microvessel. The lumen diameter, which was smaller than approximately 8 blood cells, was also taken into account.^[23]^ The mean MVD and MC counts were recorded for each case.

### 2.3. Statistical analysis

Statistical analyses were conducted using the SPSS package software, version 20. Kaplan–Meier analysis was employed to conduct survival analyses. The 2 groups were subjected to a log-rank test to ascertain any differences in survival. For continuous variables, comparisons between 2 groups were made using the Mann–Whitney *U* test, while the Kruskal–Wallis test was used for multiple groups. Spearman’s rho correlation test was employed to assess the interrelationship between variables. Receiver operating characteristic (ROC) curve analysis was employed to determine the optimal cutoff values for MVD and MCC. The patients were divided into 2 groups based on the cutoff value obtained by ROC curve analysis. Statistical significance was determined by a *P*-value ≤ .05.

## 3. Results

**Figure 1.**
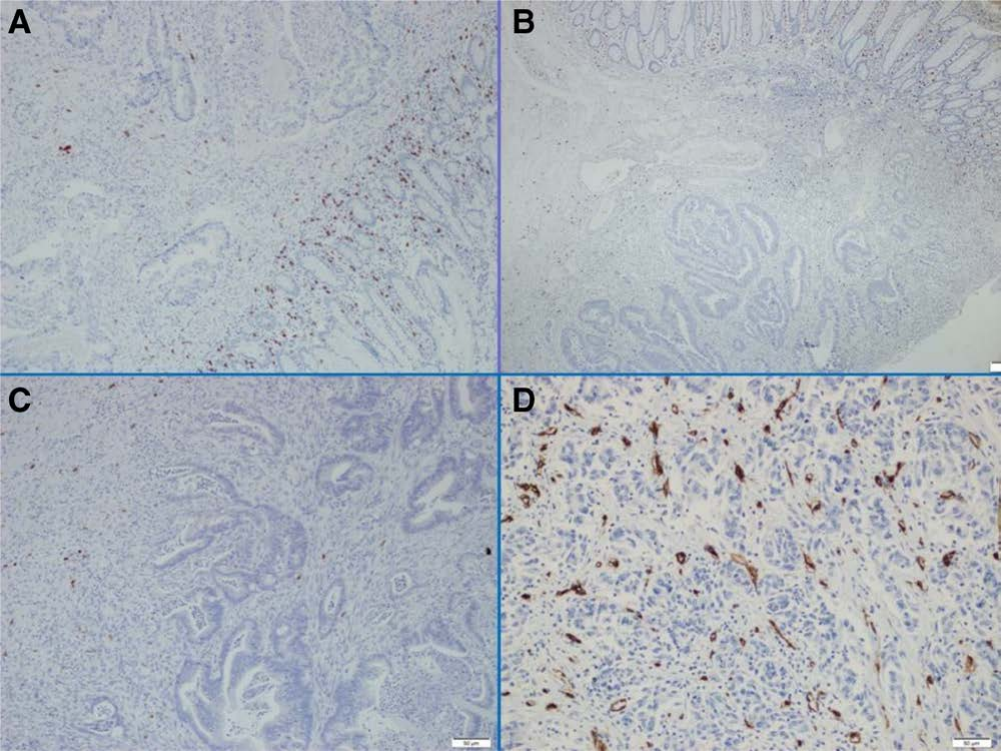
(A) CD117 positive mast cells in the mucosa adjacent to the tumor (40×); (B) CD117 positive mast cells in the tumor stroma (40×); (C) CD117 positive mast cells in the invasive side of the tumor(100×); and (D) CD34-positive microvessels in the tumor stroma (200×).

**Figure 2.**
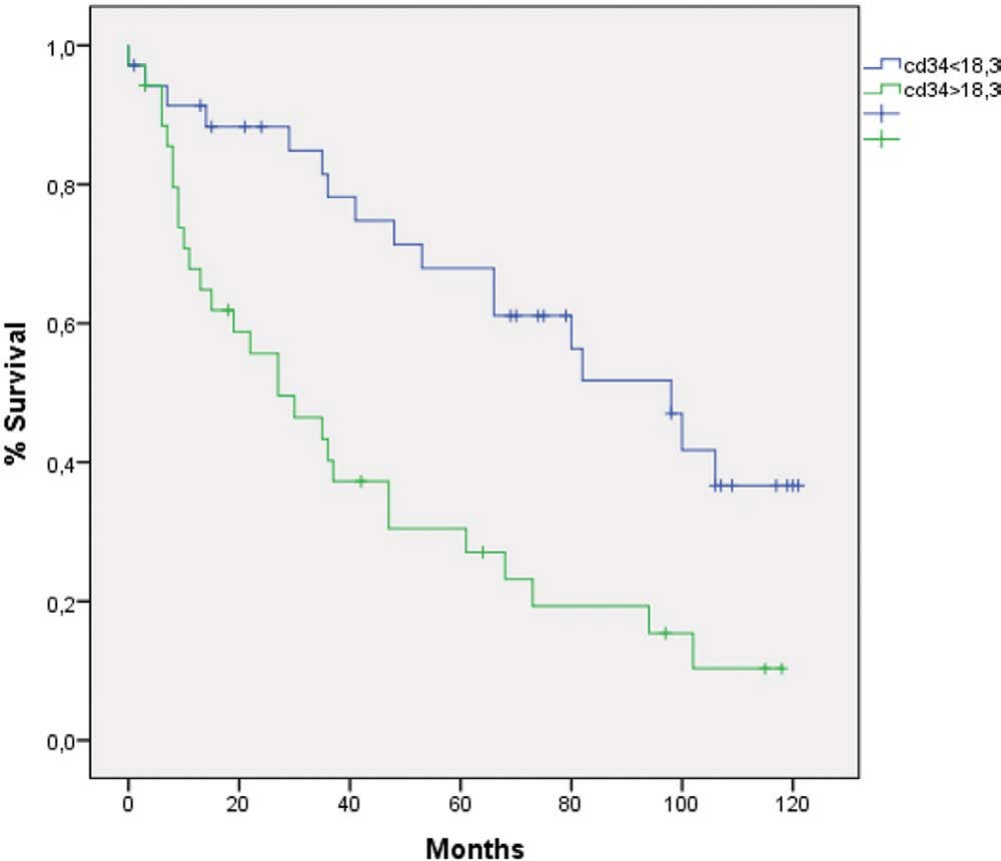
Disease-free survival according to microvascular density (*P*, .001).

**Figure 3.**
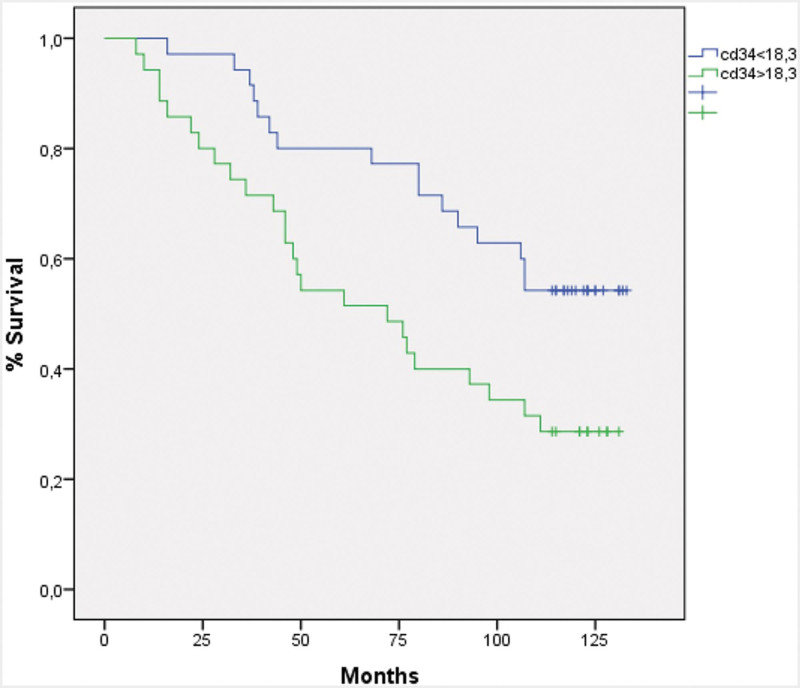
Overall survival according to microvascular density (*P*, .012).

The histopathological and demographic characteristics of the patients are presented in Table 1. A total of 94 patients who had undergone surgical treatment for CRC were included in the study. The median age of the patients was 67 years (range: 30–88 years). Of the 94 patients included in the study, 53 (56.4%) were male and 41 (43.6%) were female. Regarding tumor location, 18 patients (19.1%) had tumors in the right colon, 6 (6.4%) in the transverse colon, 39 (41.5%) in the left colon, and 31 (33%) in the rectum. Seven tumors (7.4%) were classified as well-differentiated, 76 (80.9%) as moderately-differentiated, and 11 (11.7%) as poorly-differentiated. With regard to pathological T stage, 3 cases (3.2%) were classified as pT1, 14 (14.9%) as pT2, 63 (67%) as pT3, and 14 (14.9%) as pT4. Twenty-four patients (25.5 %) had lymph node metastasis, 10 (10.6%) had perineural invasion, and 16 (17%) had lymphovascular invasion. Postoperative examinations revealed that 15 (16%) patients developed metastases. A total of 65 patients (69.1%) died during the median follow-up period of 49 months. Twenty-nine (37.2%) of the 79 patients who were initially free of recurrence or metastasis subsequently developed recurrence or metastasis. Those patients (Nine patients) who died within a short period (<4 months) after diagnosis were excluded from the survival analyses. All 85 patients included in the survival analyses exhibited a median OS of 77 months (95% CI: 56.70–95.29). Fifteen patients with metastases at diagnosis were excluded from the analysis, and the remaining 70 patients had a median DFS of 53 months (95% CI: 27.05–78.94) and a median OS of 95 months (95% CI: 70.40–119.59). The mean MCC was as follows: 12.83 (2–73.6) in the adjacent mucosa, 7.5 (0.3–23.6) in the tumor area, and 8.32 (3–29.3) in the invasive location of the tumor (Fig. 1A–C). The relationship between the prognostic factors and MCC is presented in Table 2. No significant relationship was identified between MCC and the prognostic factors (*P* > .05). MCC in the adjacent mucosa (Mmc) was found to be significantly higher than that in the tumor area (Mtm). Conversely, the MCC in the invasive area of the tumor (Minv) was significantly higher than that in Mtm, while it was significantly lower than that in Mmc (*P* < .005). The patients were divided into 2 groups based on a threshold of 6.15 for Mtm, which was calculated by an ROC curve analysis. After excluding patients who died at an early stage and those with metastasis at diagnosis, the analysis revealed 54.2% and 42.8% 5-year DFS rates for groups Mtm < 6.15 and Mtm > 6.15, respectively. The 5-year OS rates were 67.7% and 65.8% in the same groups, respectively. There were no statistically significant differences in DFS or OS between the groups (*P* > .05). The mean MVD was 18.6 (4.3–51.0) in the tumor area (Fig. 1D). The relationship between the prognostic factors and MVD is presented in Table 3. A significant association was observed between MVD and pathological T stage, lymph node metastasis, distant metastasis, and recurrence/metastasis (*P* < .05). A cutoff value of 18.3 points was used to categorize patients into 2 groups based on the number of CD34-positive stained vessel endothelial cells obtained through an ROC curve analysis. After excluding those patients who died at an early stage and those with metastasis at diagnosis, the analysis revealed 5-year DFS rates for groups CD34 < 18.3 and CD34 > 18.3 as 61.1% and 31.3%, respectively. A significant difference was observed between the 2 groups (*P* = .001; Fig. 2). The same groups exhibited 5-year OS rates of 77.1% and 51.4%, respectively. The difference was again statistically significant (*P* = .012; Fig. 3). However, no significant relationship was found between MCC and angiogenesis, as assessed by MVD, although the correlation was positive (ρ: .045, *P* = .664). MCC and MVD were found to be significantly higher in patients with metastasis at the time of diagnosis (*P* = .018 and *P* = .040, respectively).

**Table 1:** Patient and disease characteristics.

Patients	n (%)=94
Age, yr, median (range)	67(30–88)
Sex	
Male	53(56.4)
Female	41(43.6)
Localization	
Right colon	18(19.1)
Transvers colon	6 (6.4)
Left colon	39 (41.5)
Rectum	31 (33)
Histology	
Well-differentiated	7(7.4)
Moderately-differentiated	76 (80.9)
Poorly-differentiated	11(11.7)
Pathologic T stage	
pT1	3(3.2)
pT2	14 (14.9)
pT3	63(63.9)
pT4	14(14.9)
Lymph node status	
Metastatic	24(25.5)
Nonmetastatic	70(74.5)
Pathologic N stage	
N0	70(74.5)
N1	17(18.1)
N2	7(7.4)
Lymphovascular invasion	
Positive	16(17)
Negative	78(83)
Perineuronal invasion	
Positive	10(10.6)
Negative	84(76.5)
Presence of distant metastasis at diagnosis	15(16)
Recurrence/metastasis	24(34.3)
Exitus	53(56.4)
Mast cell count, mean, range	
Mast-tumour	7.5(0.3–23.6)
Mast-invasive side	8.32(3–29.3)
Mast-mucosa	12.83(2–73.6)
Microvascular density, mean, range	18.6(4.3–51.0)

**Table 2:** Mast cell counts and clinicopathologic prognostic factors.

	Mtm Mean (min–max)	Minv Mean (min–max)	Mmc Mean (min–max)	*P*
Pathologic T stage				
pT1	5.3(3–9.6)	16(9.6–29.3)	22(14–30)	>.05
pT2	5.0(0.6–13.6)	8.3(4–12.6)	12.6(5.3–41)	
pT3	6.6(0.3–23.6)	8.315(3–2.3)	12.83(2–73.6)	
pT4	8.3(0.6–22.3)	7.150(4–19)	12(3.2–31.3)	
Histology				
Well-differentiated	7(2–12.3)	11.8(9.6–12.6)	18.3(6.6–31.8)	>.05
Moderately- differentiated	6.45(0.3–23.6)	8.3(3–29.3)	12.45(4–73.6)	
Poorly-differentiated	8.3(0.6–12.6)	8.3(5.3–14.0)	14.65(2–41)	
Lymph node status				
Metastatic	7.3(0.6–22.3)	7.6(3–11.6)	13.3(3.2–43)	>.05
Nonmetastatic	6.6(0.3–23.6)	8.3(3–29.3)	12.6(2–73.6)	
Pathologic N stage				
N0	6.6(0.3–23.6)	8.315(3.0–29.3)	12.6(2–73.6)	>.05
N1	7.0(0.6–22.3)	7.6(3–13.3)	13.3(3.2–41)	
N2	9.3(2.3–17.0)	8.3(3–29.3)	13.8(4–18.3)	
Lymphovascular invasion				
Positive	9.3(0.3–22.3)	7.8(4.3–19.0)	13.8(3.2–24)	>.05
Negative	6.15(0.3–23.6)	8.3(3–29.3)	12.83(2–73.6)	
Perineuronal invasion				
Positive	8.65(2.3–13)	7.6(4.6–18.3)	8.3(3.6–36.3)	>.05
Negative	6.6(0.3–23.6)	8.3(3–29.3)	12.63(2–73.6)	
Recurrence/metastasis				
Positive	6.3(0.3–23.6)	8.3(3–23.3)	11.65(3.2–73.4)	>.05
Negative	7(0.3–22.3)	8.3(3–29.3)	13.45(2–44.6)	

Minv = c-Kit-positive mast cell count in the invasive area of the tumor, Mmc = c-Kit-positive mast cell count in the adjacent mucosa, Mtm = c-Kit-positive mast cell count in the tumor area.

**Table 3:** Microvascular density and clinicopathologic prognostic factors.

	Mean microvessel count (min–max)	*P*
Pathologic T stage		
pT1	8.4 (5.3–12.3)	.001
pT2	12.35(6–20)	
pT3	19.12(4.3–30.3)	
pT4q	26.15(11.3–51)	
Histology		
Well-differentiated	20.16(5.3–51)	.624
Moderately-differentiated	18.50(4.3–30.3)	
Poorly-differentiated	20.13(14–26.6)	
Lymph node status		
Metastatic	22.47(11.3–30.3)	.003
Nonmetastatic	17.60(4.3–51)	
Pathologic N stage		
N0	17.58(4.3–51)	.001
N1	22.89(12–30.3)	
N2	21.24(11.3–27.6)	
Lymphovascular invasion		
Positive	21.42(7.6–28)	.047
Negative	18.28(4.3–51)	
Perineuronal invasion		
Positive	21.00(15.3–26.6)	.295
Negative	18.56(4.3–51)	
Distant metastasis		
Positive	21.24(4.3–30.3)	.015
Negative	17.62(5.3–51)	
Recurrence/metastasis		
Positive	21.48(11.3–30.3)	.007
Negative	17.51(4.3–51)	

## 4. Discussion

Angiogenesis plays a role in the recovery process and embryonic development. It is also involved in the growth, progression, and metastasis of tumors.^[4]^ Angiogenesis is evaluated through the measurement of MVD, which is determined by counting the vessels that have been stained with specific antibodies within a defined area. In patients with metastatic CRC, the addition of an agent targeting angiogenesis to the treatment regimen has been shown to result in increased survival.^[24]^ Other clinical studies are currently underway to evaluate the efficacy of bevacizumab in patients who have undergone surgery for CRC.^[25]^ However, studies evaluating the relationship between angiogenesis, histopathological parameters, and prognosis have yielded disparate findings. The present study indicated a significant correlation between MVD and pathological T stage, lymph node metastasis, recurrence/metastasis, and distant metastasis. After ROC curve analysis, patients were divided into 2 groups to evaluate the relationship between MVD and survival. Patients without metastasis at diagnosis and with a high MVD demonstrated significantly shorter DFS and OS rates. In a study by Acikalin et al^[9]^, in which the authors evaluated 60 patients who had undergone surgery for CRC, a relationship was identified between MVD, recurrence, and short-term DFS. Zheng and colleagues^[10]^ conducted a multivariate analysis of 97 patients who had undergone surgery for CRC to assess the relationship between MVD and short-term OS. Nevertheless, Zygoń et al^[13]^ were unable to demonstrate a correlation between MVD and prognostic indicators in a study involving 139 patients who had undergone surgery for CRC. Yet Lindmark et al^[11]^ reported that increased MVD was associated with a longer OS. den Uil et al evaluated the prognostic significance of MVD in patients with colon cancer. The study found that MVD was associated with a poor prognosis in the stage 2 patient group and a favorable prognosis in the stage 3 patient group.^[26]^ In the present study, MVD was found to be significantly higher in patients with metastasis at the time of diagnosis. Mohammed et al^[27]^ evaluated MVD in patients with CRC. MVD has been linked to unfavorable outcomes and advanced disease stages.

In the present study, an anti-CD34 monoclonal antibody was used to assess MVD. In addition, antibodies against von Willebrand factor, CD31, and CD105 may be used to assess MVD. It should be noted that the von Willebrand factor is not present in all endothelial cells, and it is also found in leukocytes.^[28]^ The disparate findings reported by disparate studies are attributed to the discrepancies in employed antibodies and counting methodologies, in addition to the heterogeneity of the patient population.

Angiogenesis and inflammation are associated with each other. Among inflammatory cells, mast cells have been shown to play a role in tumor progression and angiogenesis. Tumor cells secrete chemotactic factors that affect the receptors expressed on mast cells. The secretion of stem cell factor (SCF) by tumor cells results in the activation of the c-Kit receptor on mast cells. The proangiogenic factors synthesized and released by the mast cells thus contribute to tumor angiogenesis.^[29]^ Animal models have demonstrated that anti-SCF and anti-c-Kit antibodies reduce tumor infiltration of marrow-originated mast cells, indicating that mast cell migration is regulated by the SCF/c-Kit axis. Consequently, the SCF/c-Kit pathway appears to be a promising target for targeted tumor therapy. There are currently phase 1–2 studies targeting mast-cell-mediated receptors and pathways in various tumors, including lung and brain cancers.^[30]^ Mast cell accumulation has been demonstrated to be associated with the rapid growth and invasion of certain tumors, including breast, lung, and prostate carcinomas.^[17–19]^ In contrast, contradictory findings have been reported in CRC regarding the relationship between mast cell density and prognosis.^[16,28–30]^ The objective of the present study was to evaluate mast cell infiltration in tissue samples from patients with colon cancer using an antibody against CD117. No correlation was identified between MCC, histopathological characteristics, and prognosis. MCC was found to be higher in patients with metastasis at diagnosis than in those with early-stage disease. The highest levels of MCC were observed in the normal mucosa adjacent to the tumor, with lower levels observed on the invasive side of the tumor and the lowest levels observed in the tumor. In addition to differentiation and activation status, local stromal factors may influence the phenotypic behavior and secretory processes of mast cells. In light of these findings, the higher number of mast cells observed on the invasive side of the tumor than in the tumor area suggests that mast cells may play a role in inhibiting tumor growth.

Gulubova et al conducted an immunohistochemical investigation of mast cell density in CRC patient samples using toluidine blue and tryptase. The study found that mast cell density was associated with poor prognosis. Additionally, the study evaluated MVD, reporting a correlation between MVD and increased mast cell density, which was associated with poor prognosis.^[20]^ Furthermore, a study by Acikalin et al^[9]^ demonstrated that this factor is associated with a poor prognosis. In this study, the authors immunohistochemically assessed mast cell density with Giemsa staining. In a study by Nielsen et al, which employed immunohistochemical evaluation of a total of 584 patients who had undergone surgical treatment for colon cancer and had been administered antibody anti-tryptase, the multivariate analysis demonstrated that MCC was an indicator of a favorable prognosis.^[21]^ In a study conducted by Mehdawi et al, the density of mast cells was evaluated using antibodies against tryptase and chymase. The results indicated that an increased mast cell density was associated with a longer OS.^[22]^

Currently, there is no standard method for evaluating mast cell infiltration in patients with CRC. Some studies have evaluated it histochemically using toluidine blue/Giemsa, whereas others have employed immunohistochemical methods. A variety of antibodies, including anti-CD117, anti-tryptase, and anti-chymase, are employed in the immunohistochemical evaluation of mast cells. The conflicting results observed in the literature regarding the association between mast cell density and prognosis in CRCs may be attributed to the use of disparate markers and methodologies for identifying mast cells, in addition to the inherent heterogeneity of the patient population, which is also evident in the present study.

## 5. Conclusion

The present study demonstrated that MVD-assessed angiogenesis was significantly related to survival and prognostic factors in patients diagnosed with CRC. Although MCC has been demonstrated to be positively correlated with MVD, the findings of the present study did not reveal any relationship between MCC and prognosis in patients with this type of cancer.

## Author contributions

**Conceptualization**: Betul Bolat Kucukzeybek, Aysegul Akder Sari, Ozcan Dere, Mine Tunakan, Yuksel Kucukzeybek.

**Data curation**: Betul Bolat Kucukzeybek, Aysegul Akder Sari, Mine Tunakan, Cigdem Dinckal.

**Formal analysis**: Betul Bolat Kucukzeybek, Yelda Dere, Cigdem Dinckal, Yuksel Kucukzeybek

**Funding acquisition**: Betul Bolat Kucukzeybek, İrfan Ocal, Aylin Orgen Calli, Cigdem Dinckal.

**Investigation**: Betul Bolat Kucukzeybek, Yelda Dere, Aysegul Akder Sari.

**Methodology**: Betul Bolat Kucukzeybek, Yelda Dere, Aysegul Akder Sari, Yüksel Kücükzeybek.

**Project administration**: Betul Bolat Kucukzeybek, Yelda Dere, Ozcan Dere.

**Resources**: Betul Bolat Kucukzeybek, İrfan Ocal, Emel Avcu, Ozcan Dere.

**Software**: Betul Bolat Kucukzeybek, Emel Avcu, Aylin Orgen Calli.

**Supervision**: Aysegul Akder Sari, Yuksel Kucukzeybek.

**Visualization**: Yelda Dere, İrfan Ocal, Emel Avcu, Ozcan Dere, Aylin Orgen Calli, Yuksel Kucukzeybek.

**Writing – original draft**: Betul Bolat Kucukzeybek, Yuksel Kucukzeybek.

**Writing – review & editing**: Betul Bolat Kucukzeybek.
